# Brain – Endocast Relationship in the Australian Lungfish, *Neoceratodus forsteri*, Elucidated from Tomographic Data (Sarcopterygii: Dipnoi)

**DOI:** 10.1371/journal.pone.0141277

**Published:** 2015-10-22

**Authors:** Alice M. Clement, Johan Nysjö, Robin Strand, Per E. Ahlberg

**Affiliations:** 1 Department of Organismal Biology, Evolutionary Biology Centre, Uppsala University, Norbyvägen 18A, 752 36 Uppsala, Sweden; 2 Department of Sciences, Museum Victoria, GPO Box 666, Melbourne 3001, Victoria, Australia; 3 Department of Information Technology, Centre for Image Analysis, Uppsala University, Lägerhyddsvägen 2, 751 05 Uppsala, Sweden; Monash University, AUSTRALIA

## Abstract

Although the brains of the three extant lungfish genera have been previously described, the spatial relationship between the brain and the neurocranium has never before been fully described nor quantified. Through the application of virtual microtomography (μCT) and 3D rendering software, we describe aspects of the gross anatomy of the brain and labyrinth region in the Australian lungfish, *Neoceratodus forsteri* and compare this to previous accounts. Unexpected characters in this specimen include short olfactory peduncles connecting the olfactory bulbs to the telencephalon, and an oblong telencephalon. Furthermore, we illustrate the endocast (the mould of the internal space of the neurocranial cavity) of *Neoceratodus*, also describing and quantifying the brain-endocast relationship in a lungfish for the first time. Overall, the brain of the Australian lungfish closely matches the size and shape of the endocast cavity housing it, filling more than four fifths of the total volume. The forebrain and labyrinth regions of the brain correspond very well to the endocast morphology, while the midbrain and hindbrain do not fit so closely. Our results cast light on the gross neural and endocast anatomy in lungfishes, and are likely to have particular significance for palaeoneurologists studying fossil taxa.

## Introduction

Since the early nineteenth century, lungfishes, or dipnoans as they are also known, have captivated researchers. Fossil material was recognized e.g. [1] even before any of the extant taxa were discovered and described [2–4]. Today there are just six species in three genera remaining in the crown group, however, their peak in diversity was undoubtedly during the Devonian (359–420 million years ago), with the number of species described from this period now approaching 100 [5, 6].

Lungfishes are a clade within the Sarcopterygii, alongside the coelacanth *Latimeria*, and tetrapods. There has long been debate about the precise phylogenetic relationships between the three groups of extant sarcopterygians [7–11], but it is now widely accepted that lungfishes are the sister taxon to the tetrapods ahead of coelacanths, using both morphological [5, 12–14] and molecular lines of evidence [15–17]. Of the surviving lungfish genera, *Lepidosiren* and *Protopterus* together constitute Lepidosirenidae, while *Neoceratodus*, the Australian lungfish, is the only remaining member of Neoceratodontidae [2, 12]. These two lungfish families are thought to have diverged during the Permian, approximately 277 million years ago [18]. The highly diverse Devonian lungfishes thus all belong to the dipnoan stem group.

As would be expected from two families that have had such long, independent evolutionary histories, there are many morphological differences between Lepidosirenidae and *Neoceratodus*, including in their nervous systems [19–23]. In fact, it is commonly stated that the brains of the lepidosirenid lungfish more closely resemble those of lissamphibians, whereas the brain in *Neoceratodus* is more like that of *Latimeria* [22, 24, 25]. Research on fossil lungfish endocasts—the mould of the internal space of the neurocranial cavity—suggests that *Neoceratodus* has retained more plesiomorphic character traits with respect to the brain and neurocranial cavity than lepidosirenids [26–28].

The brain of *Neoceratodus* was first described by Huxley [29], and later elaborated upon by numerous authors, including Bing and Burkhardt [30], Griel [31], Holmgren and van der Horst [20], Rudebeck [21] and Stensiö [26]. Even from its first description, it was noted “the brain of *Ceratodus* nearly fills the cranial cavity” [29], a theme touched upon again by Stensiö who clarified that the forebrain regions of the endocranium was “almost completely filled out by the corresponding divisions of the brain” [26].

The brain-endocast relationships of tetrapods, such as reptiles, birds and mammals, are usually considered more tightly constrained than those of fishes [32–34]. The brains of some modern chondrichthyans can occupy as little as 6% of the endocranial cavity as in the case of the basking shark *Cetorhinus* [35], and this value is astonishingly only 1% in the adults of the coelacanth *Latimeria* [36, 37]. However, in contrast, the endocast morphologies of some early diverging fossil actinopterygians show detailed representations of brain regions that suggest a close match between the brain and endocast form [38–40]. Despite having been studied for almost 140 years, the spatial relationship between the brain and the endocranial space in lungfish has never been fully described nor quantified. Through the application of microtomography (μCT) and Visualisation ToolKit (VTK) software, we herein describe aspects of the gross anatomy of the brain and labyrinth region in *Neoceratodus* and compare this to previous accounts. Furthermore, we illustrate the endocast of an extant lungfish and demonstrate the brain-endocast relationship in Dipnoi for the first time.

## Material and Methods

A formalin-fixed Australian lungfish (ANU 73578, *Neoceratodus forsteri*) was obtained from Professor Jean Joss, of the former Lungfish Research Facility at Macquarie University, Sydney, Australia. *Neoceratodus* hatchlings had been raised from eggs collected from lungfish spawning ponds at Macquarie University, Sydney, Australia (protocols approved by the Macquarie University Animal Ethics Committee, approval # 2003/001) by the lungfish research group. However, these actions were not performed by any of the authors of this study; we received the specimens already euthanized and formalin-fixed. The specimen is a very small sub-adult (juvenile) according to the developmental stages of Kemp [41], in which the skeleton is considered fully formed but the animal has not yet reached sexual maturity.

The cranium of ANU 73578 was severed behind the posterior extent of the skull before being taken through an ethanol replacement process. ANU 73578 was soaked in 2% ethanolic iodine solution for 6 weeks prior to scanning. This allows the iodine to react to differing degrees with amino acids and unsaturated carbon bonds and enhances differential tissue contrast [42, 43].

Following the iodine treatment, ANU 73578 was scanned at the Australian National University (ANU) High Resolution X-ray Computed Tomography Facility [44], with a spatial scan resolution of 16.5 microns. A polychromatic X-ray beam from the X-Tek RTR-UF225 X-ray source fired upon the specimen (mounted upon a Newport RV120PP rotation stage), and the Roper PI-SCX100:2048 X-ray camera was used to record radiographs with 2048 x 2048 16-bit pixels. Three-dimensional modeling and segmentation of the brain and neural endocast was performed using the software *VGStudio Max*, version 2.2 (Volume Graphics Inc., Germany).

Spatial overlap and surface distance between the brain and endocast were analysed using custom software implemented with the Visualization Toolkit VTK [45]. Using the segmented data as input, surface mesh representations of the brain and endocast were extracted with the marching cubes algorithm [46]. The resulting meshes were then superimposed by means of iterative closest point (ICP) registration [47]. Semi-transparent overlays and color-coded distance map visualizations were then generated from the superimposed meshes to enable qualitative assessment of spatial overlap and surface distance. Further quantitative analysis was performed by computing the symmetric mean absolute distance and the symmetric max absolute distance [48] between the superimposed meshes. Lastly, the spatial overlap between the brain and endocast was quantified by computing the Dice similarity coefficient [49]. The analytical script used for measuring the brain-endocast relationship is available and downloadable from Dryad at doi:10.5061/dryad.mt57r.

## Results

### Gross neural anatomy of *Neoceratodus*

**Fig 1:**
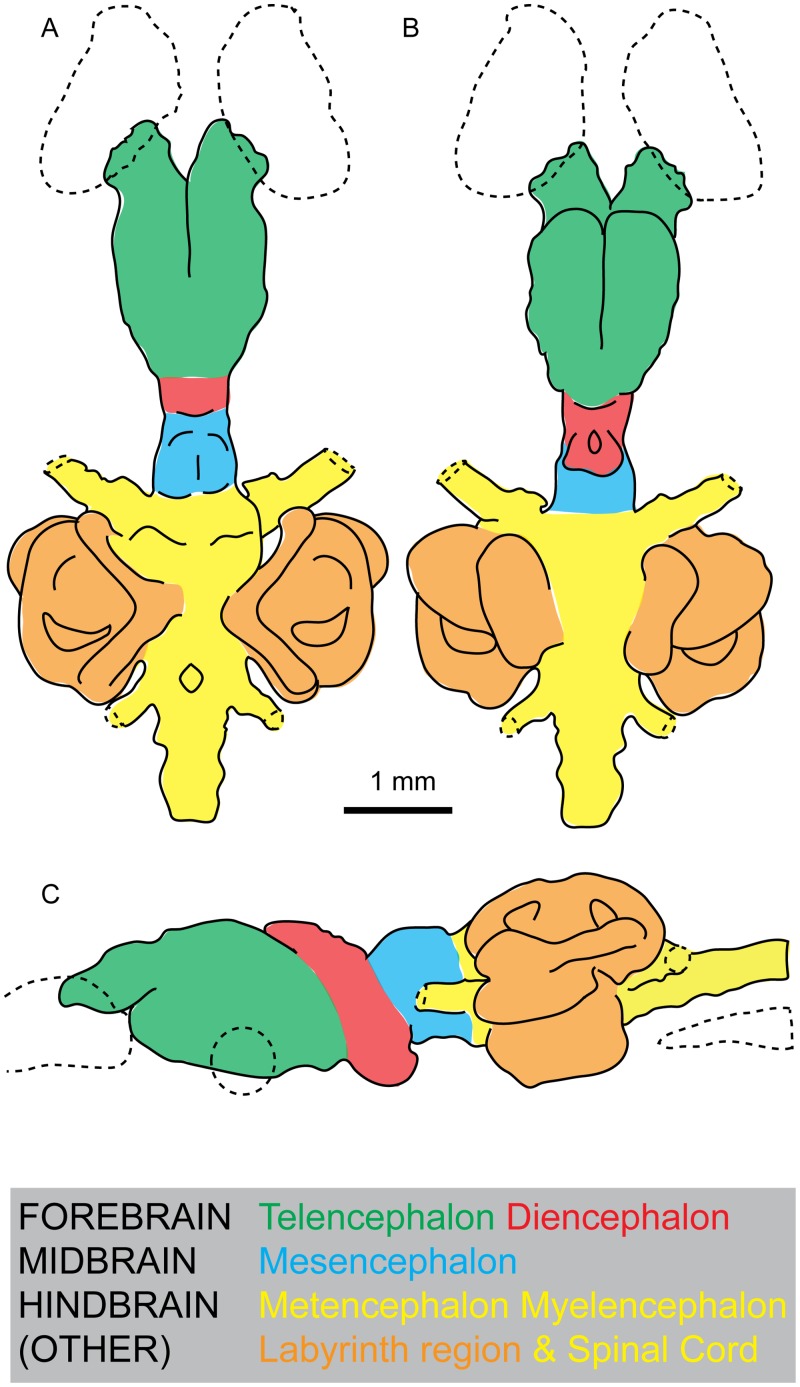
Brain regions in the Australian lungfish, *Neoceratodus forsteri*. **A**, dorsal view; **B**, ventral view; and **C**, left lateral view.

ANU 73578, a cranium of a very small juvenile, measures almost 9 mm from the anterior tip of the snout to immediately posterior of the skull, and 5.6 mm across the orbits. The brain measures almost 6 mm in length from the tip of the olfactory bulbs to the spinal cord, 3.1 mm across the widest extent of the external semicircular canals, and 1.7 mm in height from the sacculolagenar pouch to the top of the superior sinus. The forebrain (consisting of the telencephalon and diencephalon) contributes about 40% of total brain length, the midbrain (mesencephalon) 10%, and the hindbrain (metencephalon and myelencephalon) 50% (Fig 1).

### Forebrain

**Fig 2:**
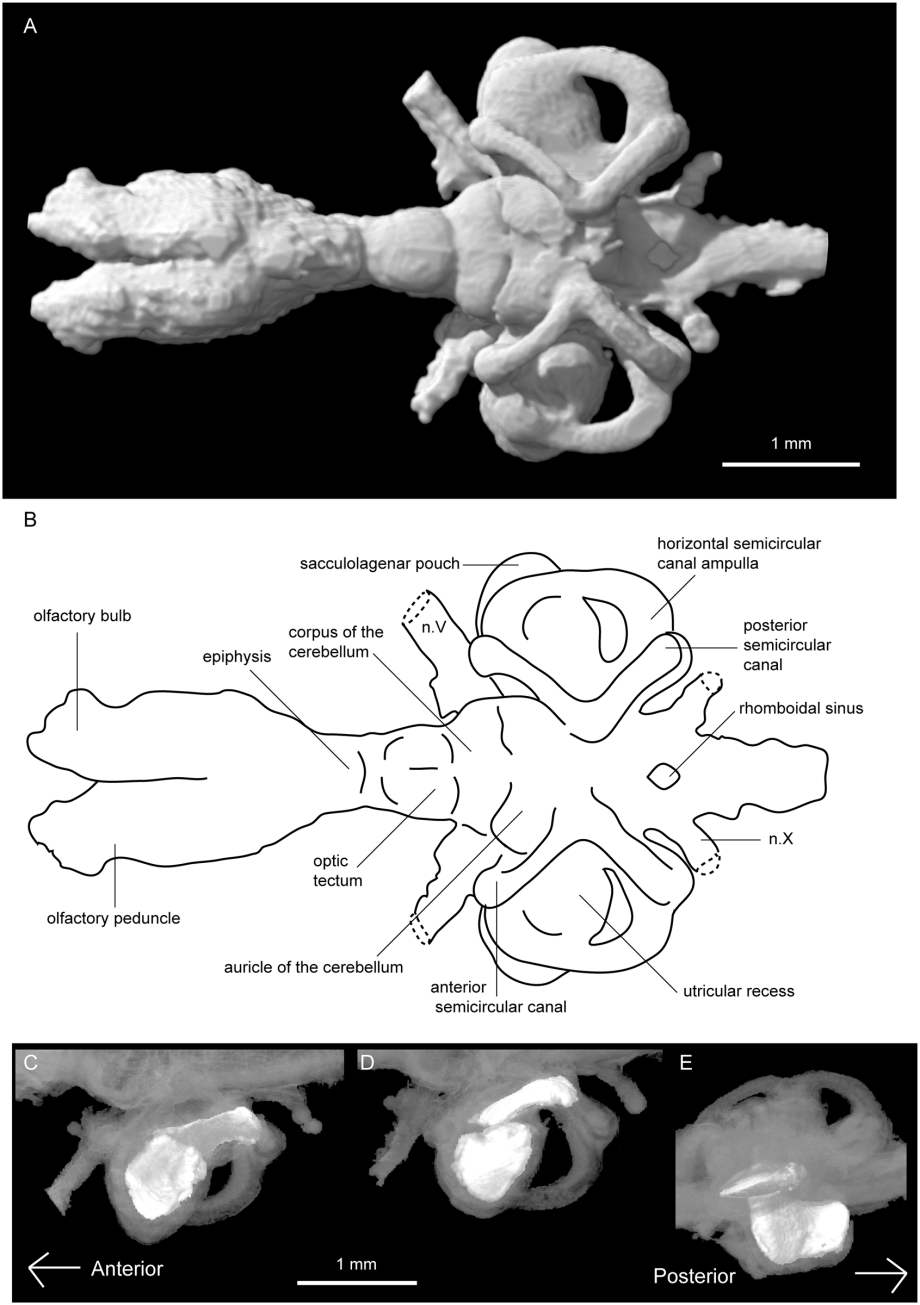
Tomographic rendering of *Neoceratodus forsteri* (ANU 73578). **A**, brain in dorsal view; **B**, interpretive drawing of the same; and left-side otoconial masses in **C**, dorsal; **D**, ventral; and **E**, lateral view. Anterior is to the left.

**Fig 3:**
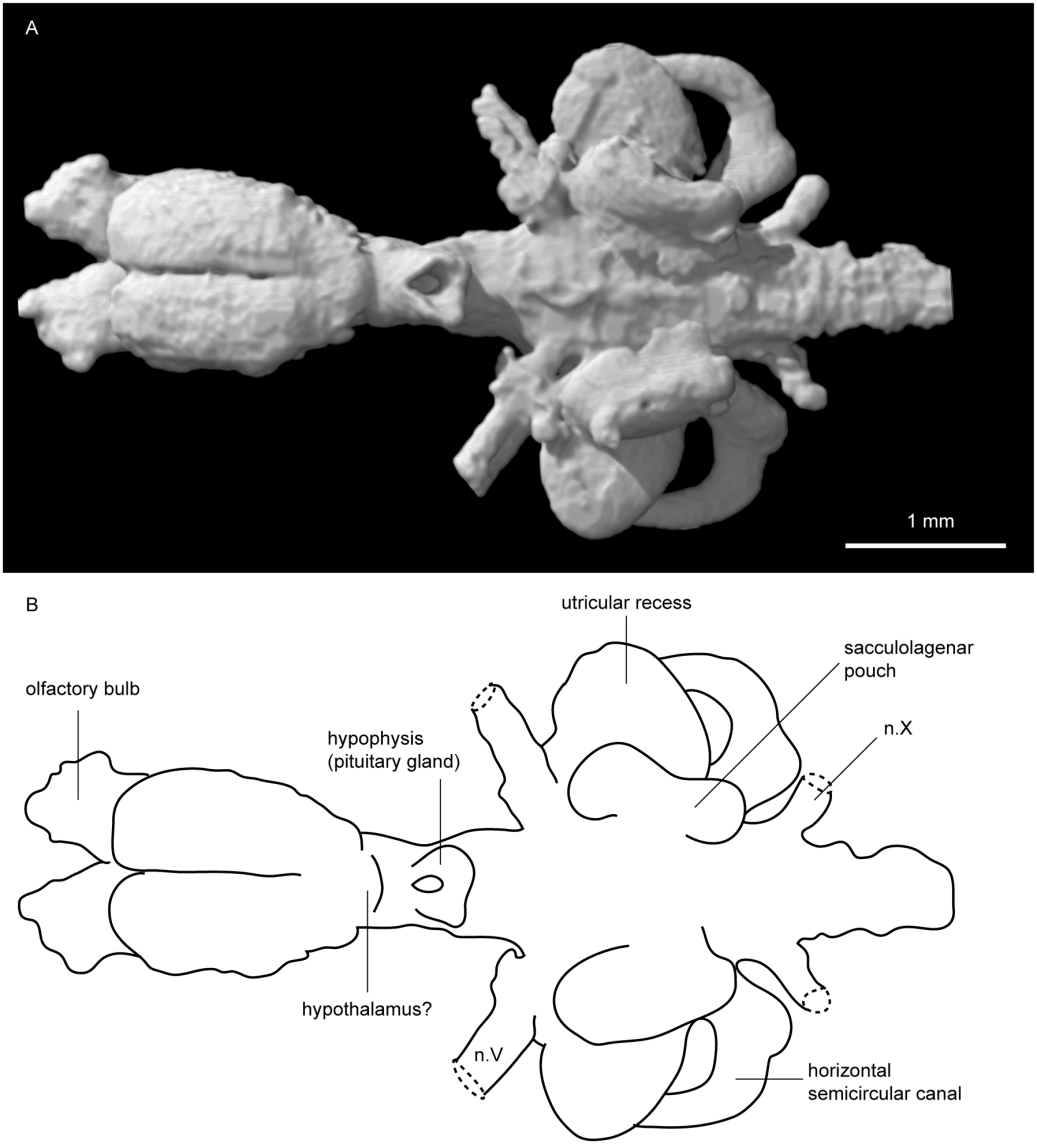
Tomographic rendering of *Neoceratodus forsteri* (ANU 73578) brain. **A**, tomographic rendering in ventral view; and **B**, interpretive drawing of the same.

**Fig 4:**
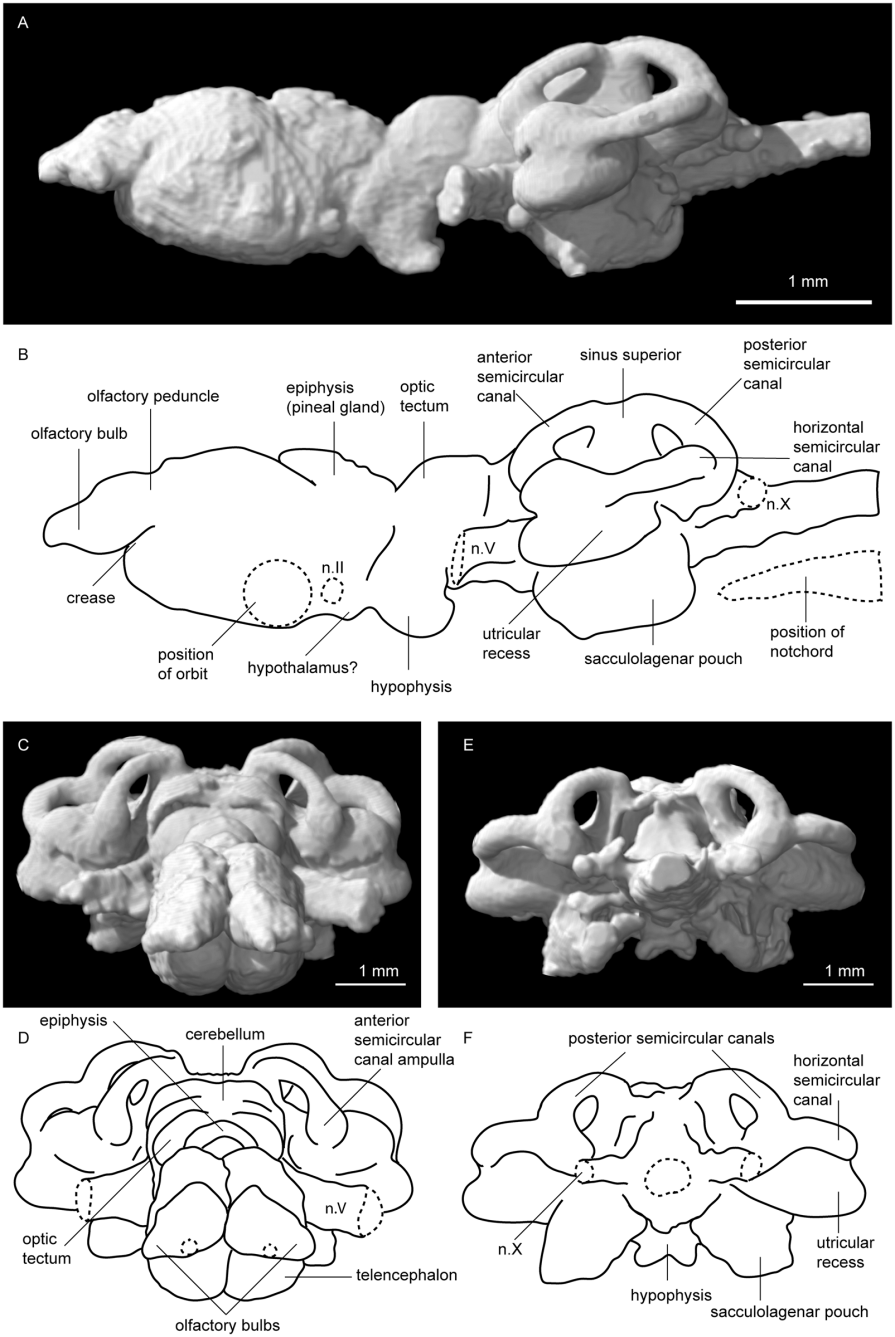
Tomographic rendering of *Neoceratodus forsteri* (ANU 73578) brain. **A**, left lateral view; **B**, interpretive drawing of the same; **C**, anterior view; **D**, interpretive drawing of the same; **E**, posterior view; and **F**, interpretive drawing of the same.

**Fig 5:**
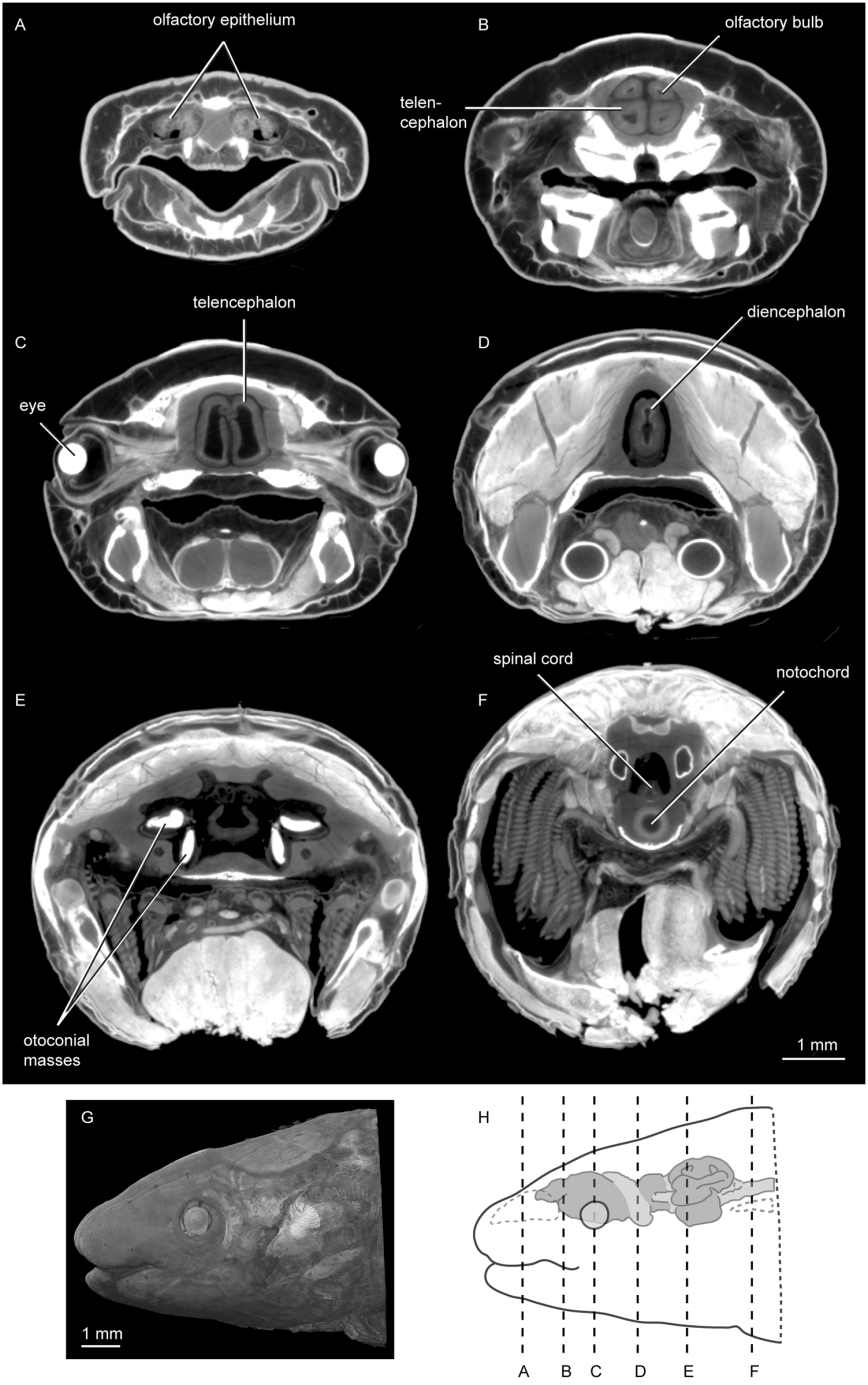
X-ray microtomographic images of iodine-treated *Neoceratodus forsteri* (ANU 73578). **A-F** in transverse view moving posteriorly; **G**, 3D rendering of whole specimen in left lateral view; and **H**, diagram showing position of slices A-F.

Anteriorly, the olfactory bulbs (Figs 2–4) are broad sub-triangular structures attaching to the anterodorsal extent of the telencephalon. They are oriented anterolaterally and have only the slightest gap between them posteromedially. The olfactory bulbs are folded over the telencephalon (Fig 5b), creating a transverse crease along their ventral edge. A slight constriction marks the olfactory peduncle, the short connection between the body of the olfactory bulbs and the telencephalon (Fig 4a and 4b).

The telencephalon is a large, bulbous structure, expanded laterally but more strongly ventrally (see green region, Fig 1). A deep groove separating the two lobes of the telencephalon runs ventrally along three quarters of their length (Fig 3). A similar groove is visible along the dorsal surface, but it is less distinct than that on the ventral surface. Near the posteroventral margin of the telencephalon, the base of nerve II (optic nerve) can be seen (Fig 4a and 4b). Just posterior to this, a gentle constriction indicates the boundary between the telencephalon and diencephalon.

The diencephalon (red region, Fig 1) is short and narrow, but deep in lateral view, extending as far dorsally but even further ventrally than the telencephalon. Its dorsal extent, the epiphysis, is gently rounded and folds over the telencephalon anteriorly, whereas as the ventral extent, the hypophysis or pituitary gland, is oriented in a posteroventral direction (Fig 4a and 4b). The hypophysis forms a sub-triangular shape in ventral view with a deep, circular depression within it. Just anterior to this, there is a slight bulge visible in ventral and lateral view near the base of the telencephalon that may represent the hypothalamus (Fig 3).

### Midbrain

The mesencephalon (blue region, Fig 1) is also very short and narrow, only slightly wider the preceding diencephalon. It is very shallow in comparison to the forebrain, and has a flat ventral surface. Dorsally, the optic tectum bulges (both dorsally and laterally) forming a convex, almost hemispherical outline, and a visible ridge separates the left and right lobes (Figs 2a, 2b and 4a–4d). Behind the optic lobes, the brain constricts strongly to form the boundary between the mesencephalon and metencephalon. This contrasts strongly with the condition in actinopterygians, where the midbrain is often greatly enlarged [50, 51]. The mesencephalon in ANU 73578 closely agrees with those described from *Neoceratodus* by Holmgren and van der Horst [20], and Northcutt [22]. By contrast, *Protopterus* and *Lepidosiren* [19, 24] lack such a distinct posterior boundary of the mesencephalon.

### Hindbrain and spinal cord

The metencephalon and myelencephalon (yellow region, Fig 1) comprise the hindbrain, and this region is significantly wider than the midbrain. The anterior extent of the metencephalon is marked by the two large trigeminal nerves (n.V) reaching anterolaterally. Dorsally, the prominent cerebellum and auricles are separated from each other by a distinct groove (Fig 2a and 2b). The corpus of the cerebellum forms an oblong shape, noticeably wider than the optic tectum proceeding it. The auricles of the cerebellum form two lobes that are even wider, and reach as far dorsally as the ventral margin of the anterior semicircular canals. Posterior to this is likely the boundary between the metencephalon and myelencephalon, but a distinct boundary cannot be traced.

The myelencephalon is long and wide, and a small opening dorsally represents the rhomboidal sinus (Fig 2a and 2b). Posteriorly, two large vagus nerves (n.X) extend posterolaterally. The dorsal edge is lower than that of the metencephalon, and relative to it the ventral edge forms an acute angle, tapering gently upwards, moving posteriorly towards the spinal cord. The spinal cord continues to narrow, and is very slightly dorsoventrally compressed in cross section (Figs 4e, 4f and 5f).

### Labyrinth Region

The labyrinth region (orange region, Fig 1), or inner ears, includes three short but robust semicircular canals with ampullar expansions present on the anterior and horizontal semicircular canals (Figs 2a, 2b and 4c, 4d). At the junction of the anterior and posterior semicircular canals, the superior sinus stands tall above the dorsal extent of the hindbrain (Fig 4a and 4b). The utriculus receives the anterior and horizontal semicircular canals, and bulges outwards in both anterior and ventral direction. Below the utriculus lies the sacculolagenar pouch, which appears almost kidney bean-shaped in ventral view (Fig 3).

Two otoconial masses are present in each inner ear (Figs 2c–2e and 5e). That in the utricular is flattened and spherical (somewhat disc-like) and lying in the horizontal plane, whereas that in the sacculolagenar pouch is sub-rectangular in outline and oriented anterior-posterior. The condition in ANU 73578 is typical for *Neoceratodus*, where the otoconial masses are smaller than those of the Lepidosirenidae, as recently shown by Challands [28].

### Brain—endocast relationship in *Neoceratodus*

**Fig 6:**
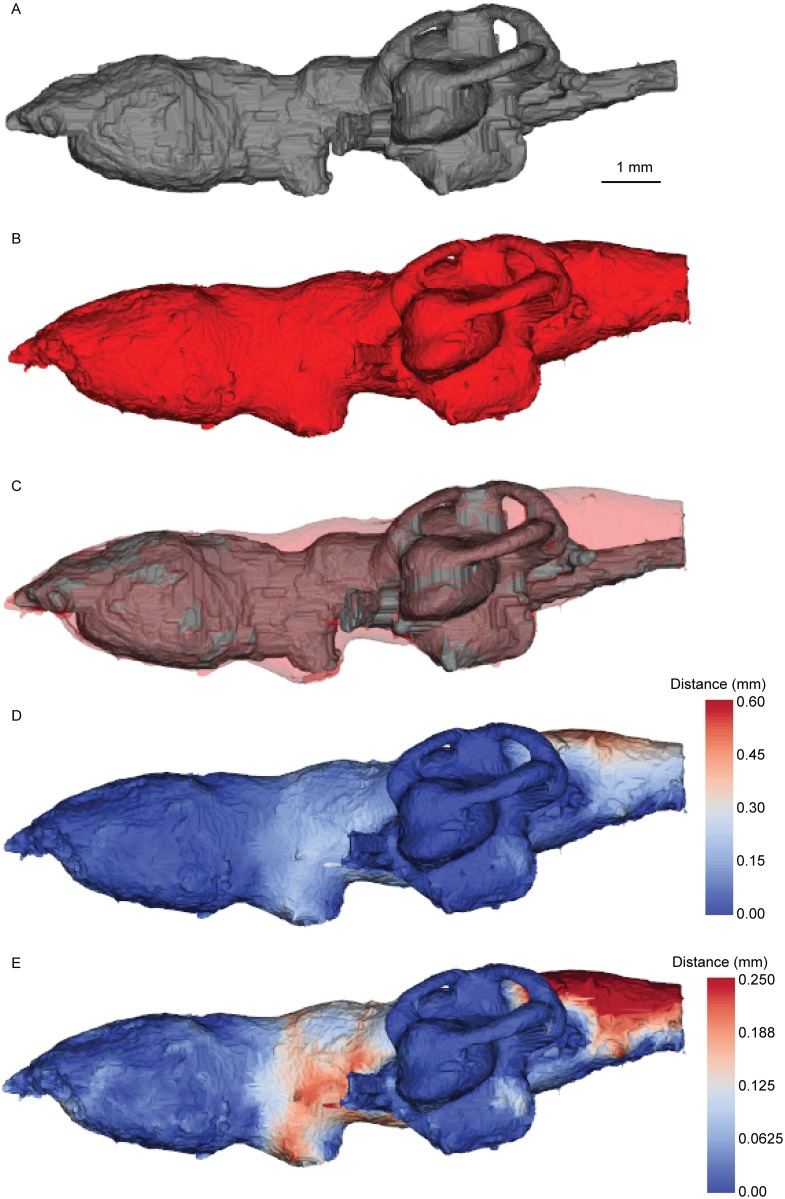
Brain-endocast spatial relationship in *Neoceratodus*, left lateral view. **A**, brain; **B**, endocast; **C**, overlay; **D**, distance map <0.590 mm; and **E**, distance map <0.250 mm. Warmest colours indicate greatest distance.

**Fig 7:**
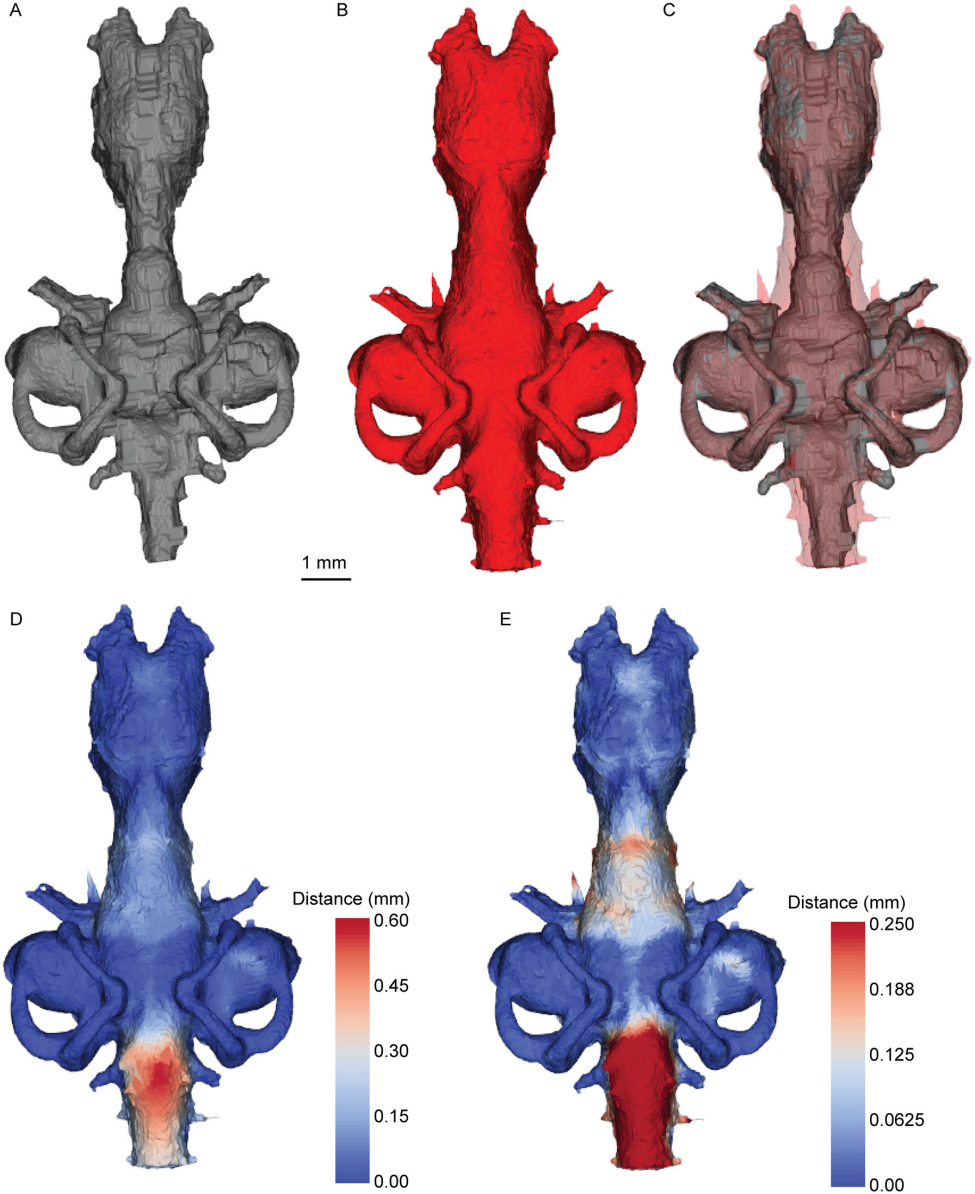
Brain-endocast spatial relationship in *Neoceratodus*, dorsal view. **A**, brain; **B**, endocast; **C**, overlay; **D**, distance map <0.590 mm; and **E**, distance map <0.250 mm. Warmest colours indicate greatest distance.

**Fig 8:**
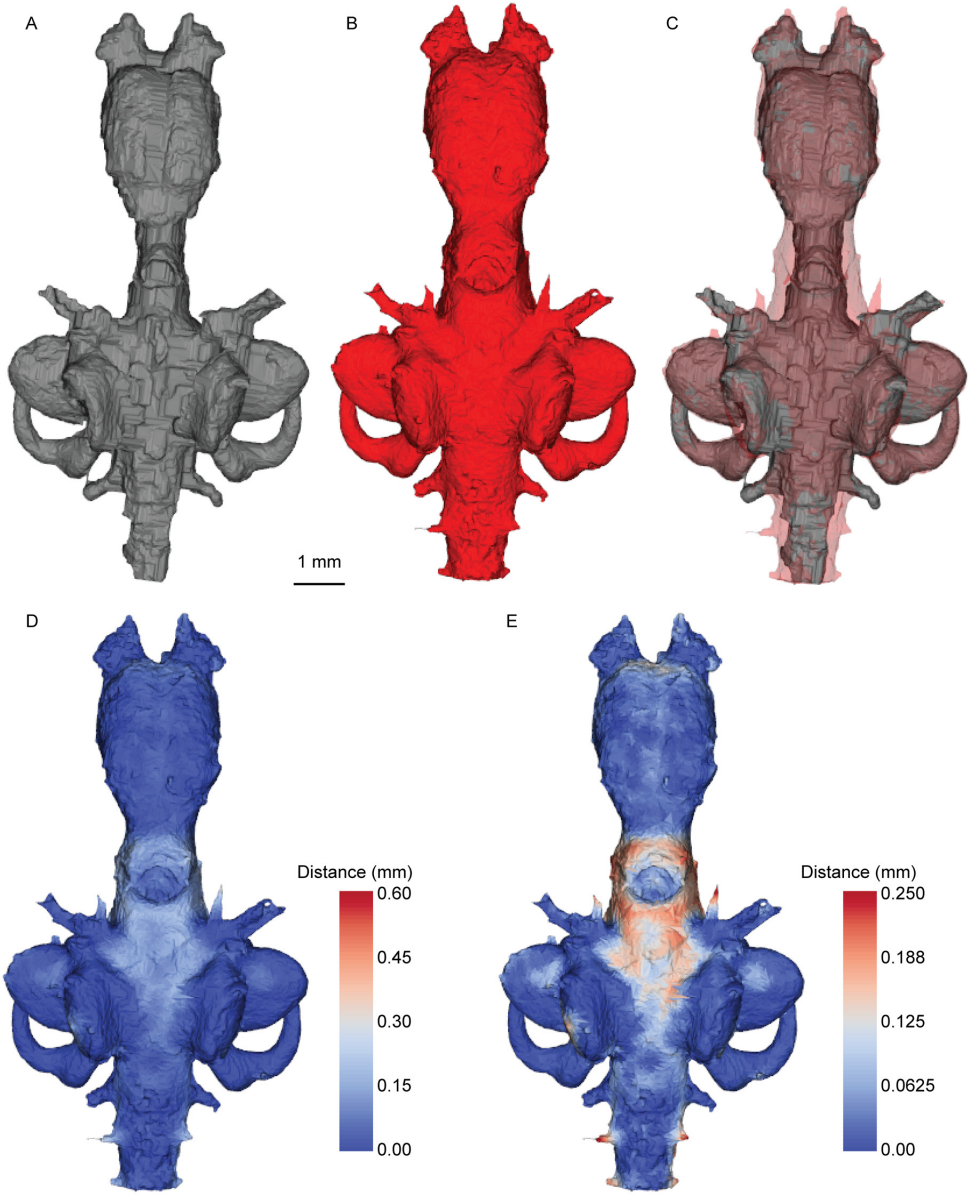
Brain-endocast spatial relationship in *Neoceratodus*, ventral view. **A**, brain; **B**, endocast; **C**, overlay; **D**, distance map <0.590 mm; and **E**, distance map <0.250 mm. Warmest colours indicate greatest distance.

Figs 6–8 show the spatial relationship between the brain and the cranial endocast in *Neoceratodus* (see also animation on Dryad doi:10.5061/dryad.mt57r). In overall shape, the morphology of the endocast (Figs 6b, 7b and 8b) closely reflects that of the brain (Figs 6a, 7a and 8a); it has a bulbous forebrain region, short and narrow midbrain, long and broad hindbrain and a prominent labyrinth region. The absolute maximum distance between any portion of the brain and its enveloping endocast is 0.59 mm, but overall the absolute mean is 0.04 mm. In the unsigned distance maps, the darkest blue colour indicates no gap between the brain and endocast, and the warmer colours indicate a greater distance. One simple overlap and two unsigned distance maps are shown in Figs 6–8. Of the distance maps, the first (Figs 6d, 7d and 8d) reveals the full distance range between the brain and endocast, the second displaying lesser distances only (0.00 mm– 0.25 mm) for greater resolution of lower values (Figs 6e, 7e and 8e). As a total percentage value, the brain of this *Neoceratodus* occupies 83% of the total endocranial space, this is in great contrast to the brain of an adult *Latimeria* [37].

From first impression, it is immediately clear how closely the brain fits the endocast in shape and volume (see also Fig 5), particularly in the forebrain and labyrinth regions, shown in darkest blue in the unsigned distance maps (little or no gap between the brain and endocast). The regions with the greatest distance to the endocast include the midbrain region, and around the spinal cord dorsally (the maximum 0.59 mm distance); this region may correspond to the supraotic cavities of *Rhinodipterus* [27] and other sarcopterygians, though we have not seen any clear evidence of endolymphatic sacs in the scan. More moderate distances, shown in light blue and always less than 0.30 mm (usually less than 0.15 mm), are present in the metencephalic region and in the sacculolagenar pouch posterolaterally (Figs 6d, 7d and 8d). In the diencephalon, the area surrounding the hypophysis is lighter indicating a distance around 0.15 mm, however, the ventral extent of this structure lies close to the endocast boundary (around 0.05 mm), as indicated by its darker colour.

## Discussion

Although lungfish have been known since the nineteenth century, it is clear we still have much to learn about their basic anatomy. While it is known that fixation using formalin can dehydrate and cause tissue to shrink [52], it is unlikely that the brain of ANU 73578 has suffered considerably from this as several portions of the brain are still in contact with the endocranial cavity housing it. We believe that the tomographic rendering of ANU 73578 represents a true likeness of the brain of this juvenile *Neoceratodus* as it was in life. It broadly agrees with previous depictions of the Australian lungfish brain in the literature, but, even allowing for intraspecific differences between individuals such as differing lengths of the forebrain [20], there remain a number of noticeable differences in the gross morphology of ANU 73578 compared to accounts in the literature. These will be discussed in further detail below.

Comparisons herein have been made principally with the reconstructions of *Neoceratodus* by Holmgren and van der Horst [20], Northcutt [22] and Retzius [53], with some additional comments concerning Lepidosirenidae [19, 22, 24]. In the hindbrain, the opening for the rhomboidal sinus is much reduced in ANU 73578 compared to those of previous illustrations where instead of a small rhomboid, a larger elongate opening exists [20, 22]; this structure is also large in Lepidosirenidae [19, 22, 24]. In the labyrinth region, the proportions and position of the utriculus and sacculolagenar pouch resemble the original illustration by Retzius 1881, Pl. XXIV [53], an image frequently reproduced by other authors [12, 27, 54–56]. However, in contrast to Retzius’ illustration, the semicircular canals are more robust and make smaller arcs in ANU 73578. This condition is also seen in other recent depictions of *Neoceratodus* [28, 57] and suggests that Retzius’ illustration may not be as accurate with respect to semicircular canal morphology. The deep groove between the corpus and auricles of the cerebellum appear much more distinct in ANU 73578 than those illustrated by Northcutt [22], however, the shape and size of these structures otherwise appear very similar. The groove separating the two lobes of the optic tectum in ANU 73578 resembles that drawn by Northcutt, but less so the very strongly marked version drawn by Huxley [29].

However, it is the forebrain of ANU 73578 that shows the most striking differences compared with previous illustrations of *Neoceratodus*. Firstly, the olfactory bulbs are situated close to one another (*cf*. more widely separated olfactory bulbs [22], Fig 3), secondly the telencephalon is more oblong than spherical in lateral view (*cf*. [20] Figs 1 and 2), and thirdly, the olfactory bulbs do not protrude far from the telencephalon on long olfactory peduncles (*cf*. [29] Fig 2; [20] Figs 1, 2). In fact, the olfactory bulbs are attached via a very short olfactory peduncle in ANU 73578. In position and orientation, the olfactory bulbs of ANU 73578 somewhat resemble the bulbs of *Protopterus* as drawn by Northcutt [22]- but unlike Fulliquet’s interpretation where the brain shows no differentiation between olfactory bulb and telencephalon body at all [19]. *Lepidosiren* ([24], Fig 3.2) closely resembles the morphology seen in ANU 73578 in position of olfactory bulbs relative to the telencephalon. This discrepancy between ANU 73578 and previously described specimens is remarkable and requires an explanation. An obvious suggestion is that the lack of elongated olfactory peduncles in ANU 73578 (and other features described above) perhaps reflects the younger ontogenetic stage of this individual. This would be consonant with Northcutt’s [22] assertion that many of the apomorphies in the lepidosirenid brain (including sessile olfactory bulbs) may have arisen as the result of paedomorphosis, the retention of juvenile traits into adulthood. The difference in shape of the telencephalon may also relate to ontogeny, or could perhaps be a consequence of the dissection and sectioning methods used on previous specimens [20].

The results of the brain—endocast surface analysis indicate that the size and shape of the dipnoan brain are closely mirrored by the cranial cavity, at least in juvenile individuals. This suggests that gross brain morphology can be inferred with high confidence from the morphology of the endocast alone in lungfish for the forebrain and labyrinth regions. The morphology of the mid- and hindbrain regions can be determined with less confidence, yet still the general proportions can be inferred. Even without knowledge of the brain-endocranial relationship, endocast form always provides a maximal limit for the size and shape of the brain region that lies within it. These results have significant implications for the field of palaeoneurology where researchers often have only the fossilized hard parts remaining with no trace of soft tissue, and are obliged to interpret brain morphology from endocast morphology alone [27, 28].

## Conclusions

Here we have presented the first virtual rendering of a lungfish brain, preserved *in situ* and undisturbed by manual dissection techniques. Features of note in this specimen of Australian lungfish, *Neoceratodus forsteri*, include olfactory bulbs connected to the telencephalon via only very short olfactory peduncles, and a more oblong telencephalon than previously depicted in the literature. Our results suggest that a number of features in the brains of lepidosirenid lungfish arose as a result of paedomorphosis.

Furthermore, we present the endocast morphology of an extant dipnoan for the first time, also describing and quantifying the endocast-brain spatial relationship. The brain of *Neoceratodus* is found to occupy more than 80% of the endocast volume. The telencephalon and labyrinth regions of the brain hold the closest fit to the endocast, while the diencephalon, midbrain (mesencephalon) and metencephalon are less so. However it is the myelencephalon region that shows the greatest discrepancy between brain and endocast morphology. Our findings contribute significantly to our current understanding of gross neural anatomy in lungfishes, and are likely to have particular significance for palaeoneurologists studying the endocasts of fossil dipnoans.
